# Pathophysiology and Imaging Findings of Bile Duct Necrosis: A Rare but Serious Complication of Transarterial Therapy for Liver Tumors

**DOI:** 10.3390/cancers12092596

**Published:** 2020-09-11

**Authors:** Satoshi Kobayashi, Kazuto Kozaka, Toshifumi Gabata, Osamu Matsui, Wataru Koda, Miho Okuda, Kenichiro Okumura, Takumi Sugiura, Takahiro Ogi

**Affiliations:** Department of Radiology, Kanazawa University Graduate School of Medical Sciences, 13-1, Takara Machi, Kanazawa 920-8641, Japan; k-kozaka@staff.kanazawa-u.ac.jp (K.K.); gabata@med.kanazawa-u.ac.jp (T.G.); matsuio@med.kanazawa-u.ac.jp (O.M.); wkoda@staff.kanazawa-u.ac.jp (W.K.); okudamiho@staff.kanazawa-u.ac.jp (M.O.); okumurak@staff.kanazawa-u.ac.jp (K.O.); tsugiura@stu.kanazawa-u.ac.jp (T.S.); ogi@staff.kanazawa-u.ac.jp (T.O.)

**Keywords:** hepatocellular carcinoma, chemoembolization, bile duct necrosis, peribiliary vascular plexus

## Abstract

**Simple Summary:**

Bile duct necrosis (BDN) is rare but serious complication of transarterial therapy for liver tumors. During development of BDN, ischemia of the peribiliary vascular plexus (PBP) induces the disruption of the bile duct epithelial protection mechanism, causing necrosis of the surrounding tissue by the detergent action of exuded bile acids, and eventually a biloma forms. Once BDN starts, persistent tissue damage to the surrounding bile duct is induced by imbibed bile acids, and portal vein thrombosis may also be observed. On CT images, BDN shows similar findings to intrahepatic bile duct dilatation, and, therefore, it is sometimes misdiagnosed. Clinicians should be aware that although BDN and biloma can usually be treated conservatively, in the presence of symptoms such as moderate or severe infection or interval growth of the biloma, prompt treatment is essential to avoid lethal abscess formation and sepsis.

**Abstract:**

Bile duct necrosis (BDN) with biloma formation is a type of ischemic bile duct injury that is one of the serious complications associated with transarterial therapies, such as transcatheter chemoembolization therapy (TACE), and radioembolization for hepatocellular carcinoma (HCC) and hepatic arterial infusion chemotherapy (HAIC) for metastatic liver cancer from colorectal carcinoma. In terms of the occurrence of BDN and subsequent biloma formation, ischemic injury to the peribiliary vascular plexus (PBP), the supporting vessel of bile duct epithelium, is thought to be intimately associated. In this paper, we first describe the anatomy, blood supply, and function of the intrahepatic bile duct, and then illustrate the pathophysiology of BDN, and finally present the imaging findings of BDN. Under the process of BDN formation, ischemia of the PBP induces the disruption of the bile duct epithelial protection mechanism that causes coagulation and fibrinoid necrosis of the surrounding tissue by the detergent action of exuded bile acids, and eventually a biloma forms. Once BDN occurs, persistent tissue damage to the surrounding bile duct is induced by imbibed bile acids, and portal vein thrombosis may also be observed. On pre-contrast and contrast-enhanced computed tomography (CT), BDN shows similar findings to intrahepatic bile duct dilatation, and, therefore, it is sometimes misdiagnosed. Differentiation of imaging findings on CT and ultrasound (US)/magnetic resonance (MR) imaging/MR cholangiopancreatography (MRCP) is important for correct diagnosis of BDN.

## 1. Introduction

Bile duct necrosis (BDN) is a subtype of ischemic bile duct injury (synonym of ischemic cholangiopathy) [1]. BDN is known to occur as a side effect of transcatheter therapies that are intended to treat hepatocellular carcinoma (HCC) and metastatic liver cancer (MLC) [2,3,4,5,6,7]. Progression of BDN causes peribiliary tissue necrosis and eventually induces biloma formation [8,9]. If biloma infection occurs by ascending cholangitis, it can form a liver abscess and cause severe sepsis or even death [10,11,12].

Bile duct ischemia is thought to be involved in the development of BDN, but there are few reports that review its pathophysiology [3,8,10]. To date, there have been almost no detailed reviews of the imaging findings of BDN after transcatheter therapy or morphological changes in the liver after development of BDN [9,13,14].

In this article, we first review the anatomy and function of the intrahepatic biliary system and its supplying vessel, the peribiliary vascular plexus (PBP). Then, we describe the pathophysiology and imaging findings of BDN after transcatheter therapy, and the changes in liver imaging over time after the development of BDN. Lastly, we describe the therapeutic approach to BDN that occurs during the course of transcatheter therapies for liver tumors.

## 2. Anatomy of the Intrahepatic Bile Duct System

**Figure 1:**
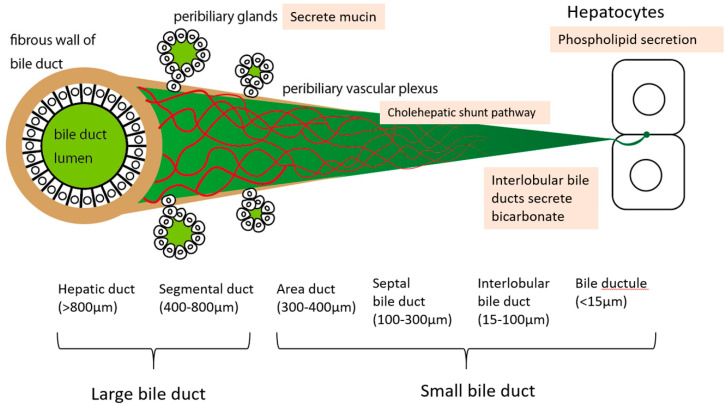
Image illustrates the size and characteristics of the intrahepatic bile ducts.

Intrahepatic biliary tracts are classified as intrahepatic large bile ducts and small bile ducts [15]. Intrahepatic large bile ducts include right and left hepatic ducts (>800 μm), segmental ducts (400–800 μm), and area ducts (300–400 μm). Small bile ducts include septal bile ducts (100–300 μm), interlobular bile ducts (15–100 μm), and bile ductules (<15 μm) (Figure 1).

Large bile ducts have fibrous walls, and there are peribiliary glands around the bile duct. A multilayered peribiliary vascular plexus (PBP) is observed around bigger bile duct than septal bile duct. Mucin secretion in the peribiliary gland, the cholehepatic shunt pathway involved in PBP, bicarbonate secretion in small bile ducts, and phospholipid secretion in hepatocytes contribute to the bile duct epithelium protection from the toxicity of bile acids.

The intrahepatic large bile duct is composed of a cylindrical epithelium. Hepatic ducts and segmental ducts have a wall consisting of dense fibrous tissue and elastic fibers. In addition, the large intrahepatic bile ducts have peribiliary glands in the wall [16]. Small bile ducts are composed of a cuboidal epithelium. The bile ducts that can be identified by needle biopsy are interlobular bile ducts and bile ducts that are smaller than interlobular bile ducts, whereas wedge biopsy can evaluate small bile ducts, such as septal bile ducts, which are larger than bile ducts that can be identified by needle biopsy.

**Figure 2:**
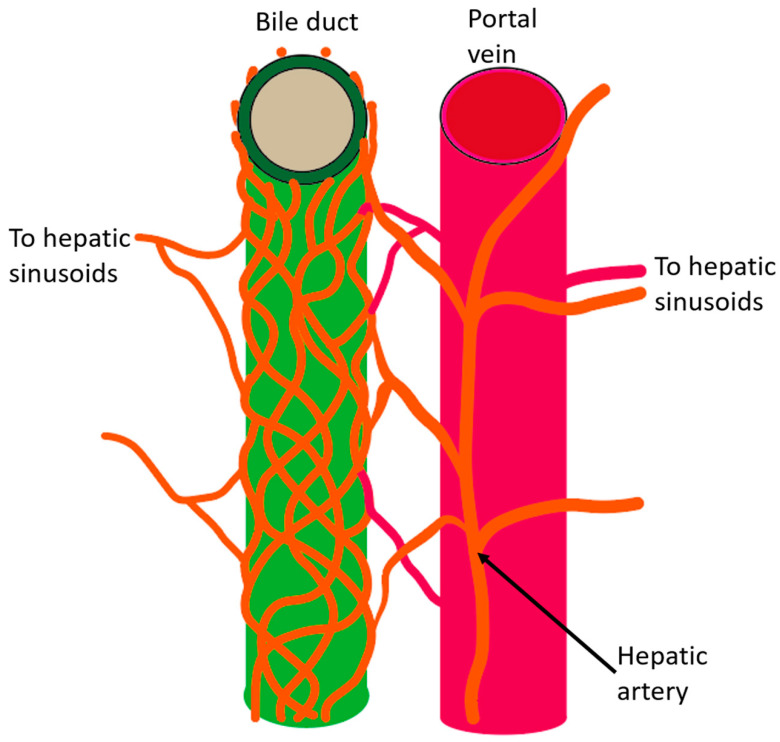
Schematic diagram of the intrahepatic bile duct and peribiliary vascular plexus (PBP). The larger intrahepatic biliary tract is supplied and nourished by a network of fine vessels called the PBP. Afferent vessels of this plexus derive from hepatic arterial branches, and this plexus drains into the portal venous system or directly into the hepatic sinusoids.

The intrahepatic and extrahepatic biliary tract is supplied and nourished by a network of fine vessels called the PBP [17,18,19]. Afferent vessels of this plexus derive from hepatic arterial branches, the gastroduodenal artery, the posterior superior pancreaticoduodenal artery, and the retroportal artery, and this plexus drains into the portal venous system or directly into hepatic sinusoids [9] (Figure 2).

**Figure 3:**
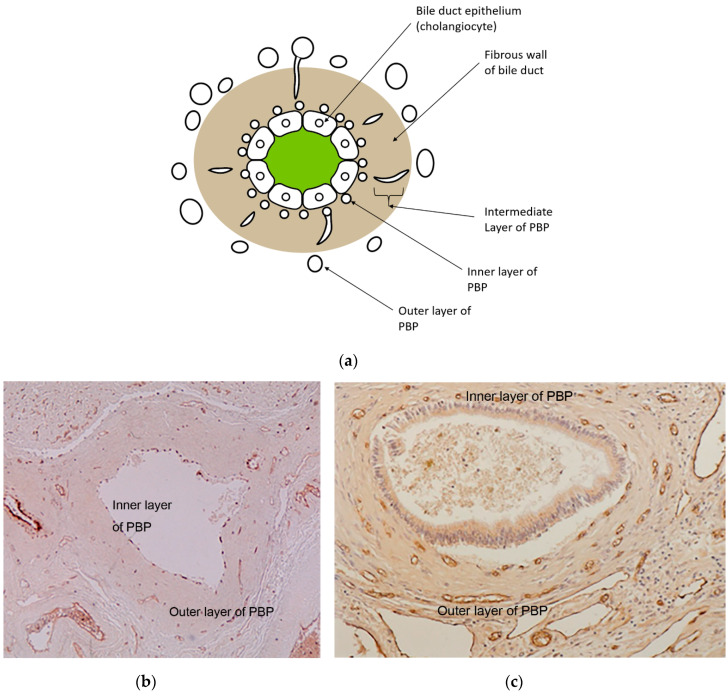
Schematic diagram and histopathologic image of PBP. (**a**) Schematic diagram of PBP. In the normal liver, the PBP around the large intrahepatic bile duct shows a three-layer pattern, i.e., the inner, the outer, and the intermediate layer. The inner layer of the PBP is observed just beneath the basement membrane of the bile duct epithelium, and the outer layer is present in the periductal tissue and is composed of arteries, veins, and capillaries. The intermediate layer vessels are in the duct walls and are presumed to connect the inner and the outer layer. (**b**,**c**) On photomicrography of the PBP in the normal liver (**b**), the inner, outer, and intermediate layer vessels of the PBP have narrow lumen and are arranged in an orderly fashion. In contrast, the PBP in the cirrhotic liver has dilated lumen and is arranged irregularly (**c**), which represents increase in collateral flow by portal hypertension. (Immunostaining of factor VIII; original magnification, (**b**): ×40, (**c**): ×100).

Histologically, the PBP is divided into an inner, intermediate, and outer layer [18]. In the large intrahepatic large bile ducts and septal bile ducts, the PBP shows a three-layer pattern, but around the smaller bile ducts, such as interlobular bile ducts, PBP shows no discernible layer formation (Figure 3). The inner layer of the PBP is a layer of capillaries that is observed just beneath the basement membrane of the bile duct epithelium (cholangiocytes). The outer layer is present in the periductal tissue and is composed of arteries, veins, and capillaries. The intermediate layer vessels are in the duct walls and presumed to connect the inner and the outer layer.

The PBP plays a fundamental role in supporting the secretory and absorptive functions of the biliary epithelium [20]. In the cirrhotic liver, the PBP works as the collateral pathway of portal hypertension and shows marked dilatation and an increase in numbers [18]. In contrast, after repeated transcatheter chemoembolization therapy (TACE) for the treatment of HCC in the cirrhotic liver, the PBP shows a decrease in numbers, especially in the inner layer vessels [21].

## 3. Function of the Bile Duct and the Significance of the PBP

Although the main bile production site is hepatocytes, around 40% of the total bile production is of ductal origin in humans [22]. Secretory functions are mainly performed by cholangiocytes (synonym of “bile duct epithelium”) lining the interlobular, septal, and major bile ducts, as they express the appropriate ion transport systems and hormone receptors in polarized domains of the plasma membrane [15].

Bile acids (BAs) are major organic solutes in the bile and are involved in several important functions in the liver and intestine. BAs are steroid compounds that are biosynthesized by the liver from cholesterol. The main action of BAs is to activate lipases and form micelles along with dietary lipids to promote their absorption, but they also have an important role in regulating cholesterol metabolism via the conversion of cholesterol to BAs. Discharged from the bile ducts to the duodenum, most of the BAs that reach the ileum are reabsorbed by the ileum mucosa and repeat the enterohepatic circulation.

BAs may disrupt cell membranes through their detergent action on lipid components, and they can promote the generation of reactive oxygen species and eventually cause cellular necrosis and apoptosis [23]. There are some mechanisms to protect the bile duct epithelium from the aggression of BAs (Table 1, Figure 1).

Physiologically, the balance of BA influx and efflux in cholangiocytes is maintained by BA transporters to avoid accumulation of toxic BAs. BAs are absorbed by cholangiocytes and subsequently excreted into the PBP, and then followed by reabsorption by hepatocytes and re-excretion in the form of bile. This recycling of BAs between hepatocytes and cholangiocytes through PBP is referred to as the cholehepatic shunt pathway [24].

In large bile ducts, the peribiliary glands, which are located in the bile duct wall, secrete mucin into bile duct lumen and are involved in protecting the bile duct epithelium [16]. In contrast, the small bile ducts do not have peribiliary glands and do not have mucin secretion capacity. In addition, the PBP is also underdeveloped in small bile ducts, so protection by the cholehepatic shunt pathway is not expected. Therefore, in small bile ducts, bile duct epithelial cells secrete bicarbonate to protect bile duct epithelial cells from highly cytotoxic BAs, which is called the biliary HCO_3_^−^ umbrella [25]. Anion exchanger2 (AE2) is involved in this mechanism. AE2 expression is down-regulated in primary biliary cholangitis (PBC), a disease caused by the destruction of small bile ducts [26]. The phospholipids, which are secreted by hepatocytes, provide another bile duct epithelial protection mechanism [27]. Phospholipids are secreted into bile in humans by the multidrug resistance 3 (Mdr3) P-glycoprotein, which is located in the canalicular membrane of hepatocytes.

Under normal circumstances, the detrimental effects of BAs are largely antagonized by phospholipids and the formation of mixed micelles in bile. However, if the secretion of phospholipids is depressed, the toxicity of bile salts is not neutralized, which might induce structural and functional injury to the cellular membranes of the bile duct epithelium and cause cellular necrosis and apoptosis [23].

## 4. Pathophysiology of BDN after Transcatheter Therapy

BDN is one form of ischemic bile duct injury [1]. Ischemic bile duct injury may show the following pathologic changes: bile duct necrosis, bile leakage, biloma, and bile duct fibrosis or stenosis. Within ischemic bile duct injuries, BDN develops predominantly where there is an abrupt and complete interruption of arterial blood supply, such as hepatic artery thrombosis in a liver transplant recipient [28]. In contrast, bile duct stenosis develops where there is progressive injury to the hepatic arterioles, such as after repeated hepatic arterial infusion of anticancer drugs (HAIC).

TACE and HAIC have been used since the 1980s to treat advanced hepatocellular carcinoma (HCC) and liver metastasis from colorectal cancer [2,6]. BDN has been reported as a serious complication of these treatments [2,3,6,7].

Although biliary strictures are common biliary complication of ischemic bile duct injury, BDN and subsequent intrahepatic biloma formation are also important complications of transcatheter therapy for hepatic tumors [1,10]. In one autopsy study, the incidence of bile duct necrosis after transcatheter arterial chemoembolization (TACE) for HCC was 9% [3].

HAIC is a reasonable drug delivery system for patients with intrahepatic metastases and advanced HCCs. However, one study reported that bile duct abnormalities developed in 57% of patients either during HAIC or 1 to 12 months after treatment [29]. In addition, the incidence of BDN after HAIC for treatment of colorectal metastatic liver tumor was reported to be 1.8% [6].

**Figure 4:**
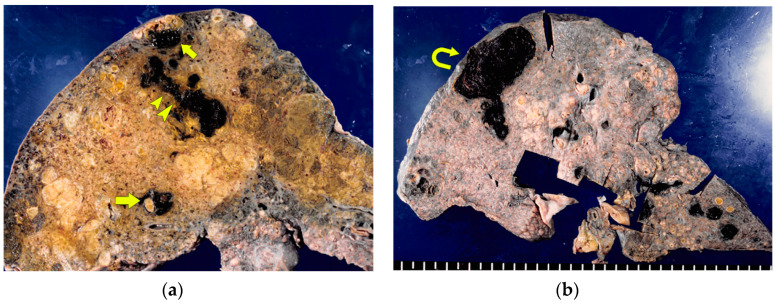
Bile duct necrosis (BDN). (**a**) Photograph of a gross specimen of the cut surface of the liver. There are round-shaped (arrow) and linear (arrow heads) dark green lesions in the right lobe of the liver. These images represent the cross-section and longitudinal cross-section of the necrotic bile ducts. (**b**) Photograph of a gross specimen of the cut surface of the liver. The necrotic bile duct, which shows a dark green tubular structure, is consecutive to the biloma, which shows a large dark green cystic mass like a lesion (curved arrows).

In macroscopic specimens, BDN is recognized as a dark green, circular or tubular area centered on the portal tract in the cross-section of the liver (Figure 4a) [3]. In BDN with biloma formation, a necrotic bile duct showing dark green tubular structures is consecutive to biloma formation (Figure 4b).

**Figure 5:**
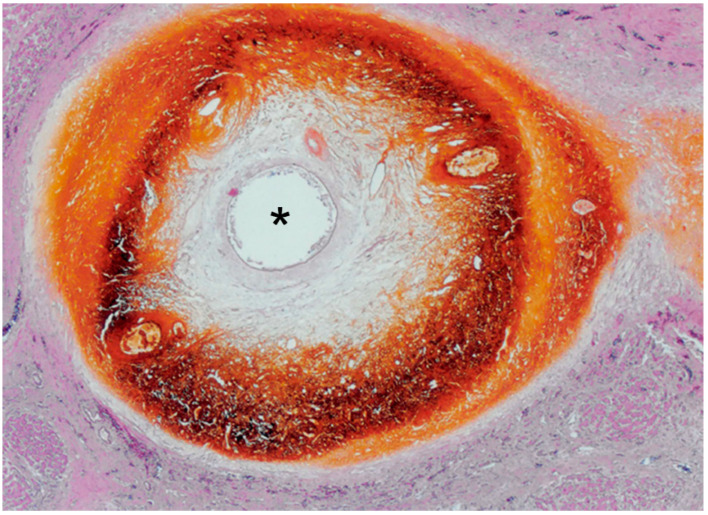
Photomicrograph of BDN. Peri-bile duct tissue, including the portal tract contents and surrounding hepatic parenchyma, is imbibed by the bile, which shows a dark brown color, and shows coagulation necrosis. The bile duct is located at the center of the necrotic area and the lumen is patently open (*). (H&E stain; original magnification, ×40.).

Histopathologically, peri-bile duct tissue, including the portal tract contents and surrounding hepatic parenchyma, are imbibed by bile and show necrotic change, including coagulation necrosis and fibrinoid necrosis (Figure 5) [3,9,10].

**Figure 6:**
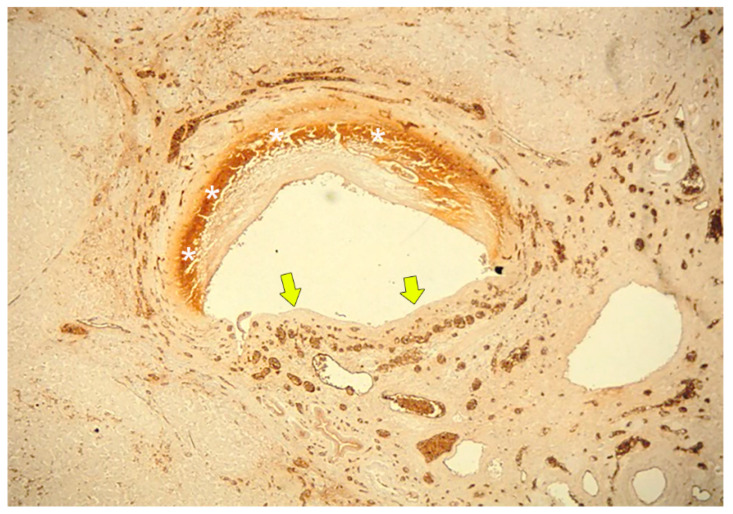
Photomicrograph of BDN. About the half of the bile duct wall shows necrotic change and shows a dark brown color (*). The inner layer of the PBP totally disappears beneath the basement membrane of the bile duct epithelium (arrows). In contrast, the outer layer of the PBP is dilated and increased in number (arrowheads). (Immunostaining of factor VIII; original magnification, ×40.).

Inflammatory cell infiltration is also observed in peri-bile duct tissue [30]. The bile ducts are located at the center of the necrotic changes, and the lumen of the bile ducts is patently open. Therefore, the bile produced in the distal liver is excreted through the open lumen of the necrotic bile duct in the area of BDN and flows into the bile ducts of the proximal portion of the liver. This is strongly related to the persistent exacerbation of bile duct necrosis, which leads to biloma formation. Immunostaining of the vascular endothelium shows that PBP vessels of adjacent to the BDN area have disappeared or are markedly reduced (Figure 6) [3].

Generally, in the cirrhotic liver, the PBP is dilated and increased in both the inner and outer layers compared to the normal liver [3]. However, repeated TACE or HAIC can induce development of vasculitis, which can lead to deterioration (reduction or loss) of PBP vessels, as well damage to the hepatic arterial branches. For a PBP that has been devastated by TACE, further TACE will result in disruption of PBP blood flow and ischemia and injury to the bile duct epithelium [3,10]. Similarly, repeated HAICs can also lead to bile duct epithelial injury due to the deterioration of the PBP. The decrease in or loss of PBP blood flow causes dysfunction of bile duct epithelial cells and reduces the secretion of phospholipids from hepatocytes, which work to protect bile duct epithelial cells [27]. Therefore, bile duct epithelial cells are unable to protect themselves from the detergent action of BAs, leading to cell necrosis and apoptosis. Reduced PBP blood flow also causes dysfunction of the cholehepatic shunt pathway, which transports BAs absorbed by cholangiocytes to the hepatocytes [24]. Consequently, injury to the bile duct epithelium becomes more severe.

**Figure 7:**
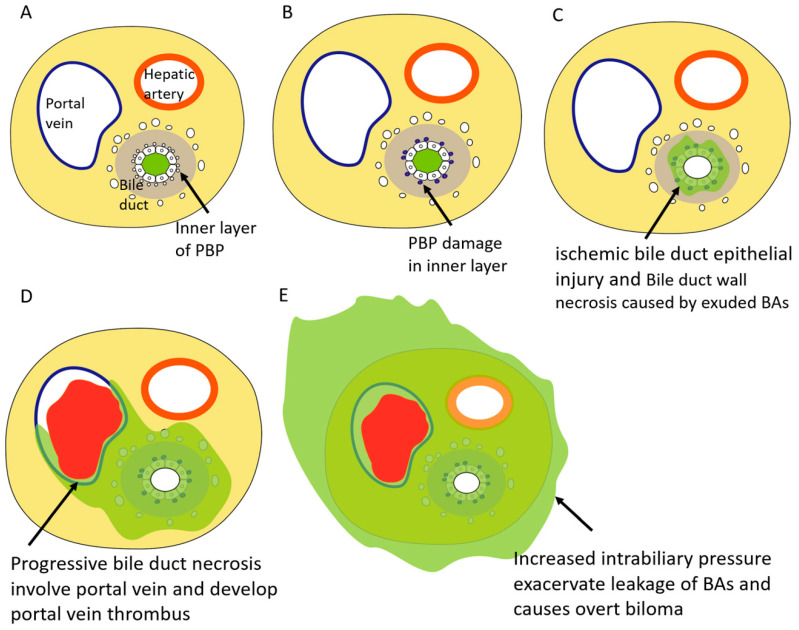
Schematic diagram of the development of BDN and subsequent biloma formation. In liver cirrhosis, both inner and outer vessels of PBP show dilatation and increased in number (**A**). After repeated TACE, the inner layer of PBP vessels are damaged and decreased in number (**B**). Once ischemic bile duct epithelial injury is caused by PBP disorder, toxic bile acids (BAs) exude to the bile duct wall and show necrotic change in and around bile duct, which is the early stage of BDN (**C**). If the progressive necrosis of peri-bile duct tissue caused by exuded BAs involves the portal venous branch, portal vein thrombosis can be easily developed (**D**). If ischemic bile duct injury downstream of the BDN site occurs, stenosis of the downstream bile duct may induce intrahepatic bile stasis, which causes an increase in intrabiliary pressure at the bile duct epithelial injury site, which leads to increased leakage of BAs into the surrounding tissues and causes overt biloma formation (**E**).

Sakamoto et al. stated that the interaction between two factors is important for the development of BDN (Figure 7) [10].

One factor is bile duct epithelial injury caused by a PBP disorder in BDN site. The other factor is the stenosis of the downstream bile duct caused by ischemic bile duct injury downstream of the BDN site [3,10]. Stenosis of the downstream bile duct may induce intrahepatic bile stasis and cholangitis. Bile stasis causes an increase in intrabiliary pressure at the bile duct epithelial injury site, which leads to increased leakage of BAs into the surrounding tissues through the bile duct epithelium and extensive necrosis of structures in the portal tract and adjacent hepatocytes due to the toxic detergent action of BAs [31]. Consequently, overt BDN which is recognized on diagnostic imaging is developed.

BDN is less frequent in liver transplantation. This is due to the fact that retrograde blood flow from the portal system maintains PBP blood flow, even after the disruption of hepatic arterial blood flow toward the PBP in the case of arterial blood flow impairment during liver transplantation [32,33,34]. In contrast, in TACE and HAIC, bile duct epithelial injury is more pronounced because the PBP itself is impaired and blood flow within the PBP is intercepted [3,35]. TACE for liver metastases is more likely to produce BDN than TACE for HCC [36]. There are two reasons for this. One reason is that, compared to hypervascular HCC, liver metastases are hypovascular, and drug accumulation in the peritumoral normal liver is greater than drug accumulation within the tumor. The other reason is that the background liver condition in metastatic liver cancer is usually normal, and the normal liver is prone to PBP injury because the PBP in the normal liver is not as dilated and enlarged as observed in cirrhotic liver [3].

**Figure 8:**
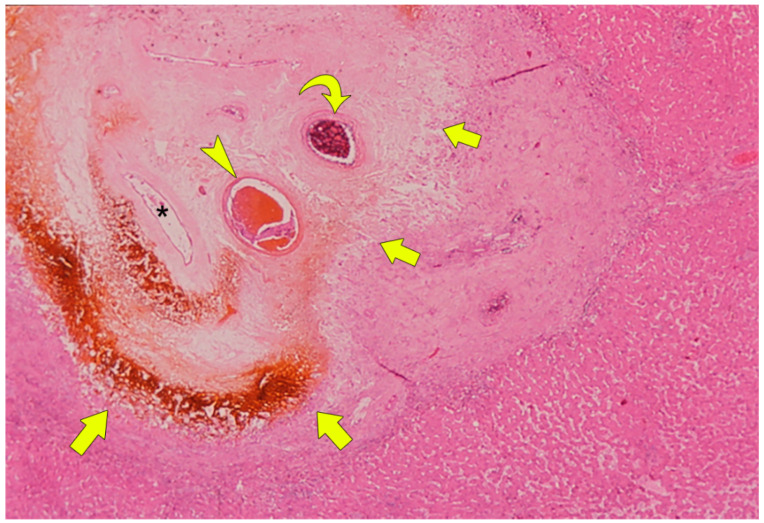
Photomicrograph of BDN. Coagulation necrosis is observed within the portal tract (arrows). The lumen of the necrotic bile duct is open (*). Thrombosis is observed in the portal venous branch (arrowhead). Embolic material is observed in the hepatic arterial branch (curved arrow). (H&E stain; original magnification, ×40.).

Damage to the bile duct epithelium by BAs causes bile to leak into the peri-bile duct tissue, leading to progressive necrosis of the peri-bile duct tissue. At this moment, the portal vein that runs along the bile ducts is easily damaged because of its thin wall, and portal vein thrombosis can easily be developed (Figure 8) [36].

As a result, portal venous blood flow to the periphery of the liver is disrupted at the BDN site. In contrast, the hepatic artery running along the necrotic bile duct is less vulnerable to damage than the portal vein because of the thick fibromuscular layer of the arterial wall. Therefore, hepatic artery occlusion occurs at a more advanced (later) stage of BDN than portal vein occlusion.

Bile inflow from the peripheral part of the liver is persistent in bile ducts that have developed BDN. However, bile duct stenosis is present in the bile ducts downstream portion of the BDN site. This causes increased intra-bile duct pressure and continuous leakage of BAs at the site of bile duct epithelium damage. Therefore, necrobiotic change in the periductal tissue adjacent to the damaged bile duct persistently expands, even after discontinuance of therapeutic interventions such as TACE and HAIC, which damage the bile duct and eventually form a biloma. When the portal tract and its surrounding components, such as the canal of Hering and hepatic progenitor cells, become necrotic due to the toxic effect of BAs exude from BDN, the liver cannot regenerate in this area and gradually shows atrophy. This may have a synergistic effect on the hepatic parenchymal atrophy caused by portal blood flow damage due to the exuded BAs.

Intrahepatic bile duct dilatation and cholestasis are not seen upstream of the BDN site because the bile duct lumen remains open in the bile duct necrosis area. However, at the site of BDN, occlusion of the portal vein and hepatic artery occurs, which results in peripheral hepatic atrophy.

In the case of retrograde infection from the gastrointestinal tract, the BDN site and/or biloma become a liver abscess, which may result in lethal sepsis [10,11,12].

## 5. Imaging Findings of BDN

BDN is usually depicted on CT scan for closer examination of laboratory data abnormality, i.e., marked elevation of serum alkaline phosphatase (ALP) either with or without increased serum bilirubin during the course of repeated TACE or HAIC [27,33]. However, it is difficult to make an accurate diagnosis of BDN on CT without sufficient knowledge, because it shows similar findings to that of intrahepatic bile duct dilatation, especially at the early stage.

Here, we present for the first time the CT findings of BDN. We also describe the findings of other imaging modalities that may be useful in confirming the imaging diagnosis of BDN. Finally, we describe the subsequent CT course of the liver after the occurrence of BDN.

• CT findings of bile duct necrosis

**Figure 9:**
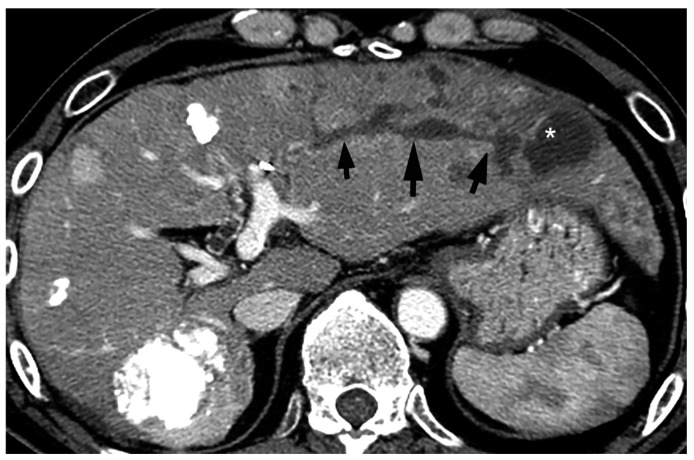
Portal phase image of contrast-enhanced CT of BDN and the biloma after transarterial therapy. A hypodense tubular structure (arrows) is connected to the large cystic lesion (*) in the lateral segment of the liver. The tubular structure is BDN of the anterolateral segment (S3) and the cystic lesion is biloma.

On pre-contrast and contrast-enhanced CT (CE-CT), BDN shows a linear hypodense structure along with a portal tract which looks like “intrahepatic bile duct dilatation” (Figure 9) [10,36]. The diameter of the lesion may show irregularity and might show findings similar to the “beaded-like appearance” observed in primary sclerosing cholangitis. Often, this finding is misdiagnosed as intrahepatic bile duct dilatation because the reason for the CT examination is a close examination of the cause of liver dysfunction, including increased serum bilirubin.

Structures with similar imaging findings in the liver after TACE have been reported under the following names: branched biloma [12], focal or multiple intrahepatic bile duct dilatation [11], and bile duct dilatation with extravasated bile collection along the portal tract [37]. Each of these is the same pathological condition as BDN.

**Figure 10:**
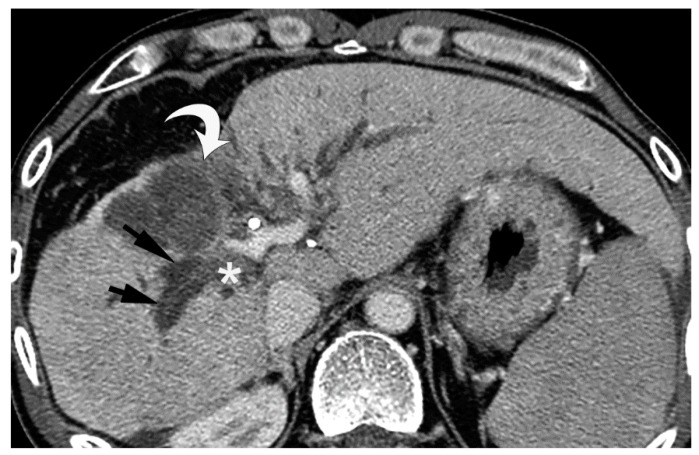
Equilibrium phase image of a contrast-enhanced CT of BDN. A hypodense tubular structure (arrows) is observed in the hepatic hilum of the right lobe. Enhancement of the right main portal branch is discontinued (*), which represents thrombotic obstruction of the portal vein caused by BDN. (Curved arrow is the biloma.).

**Figure 11:**
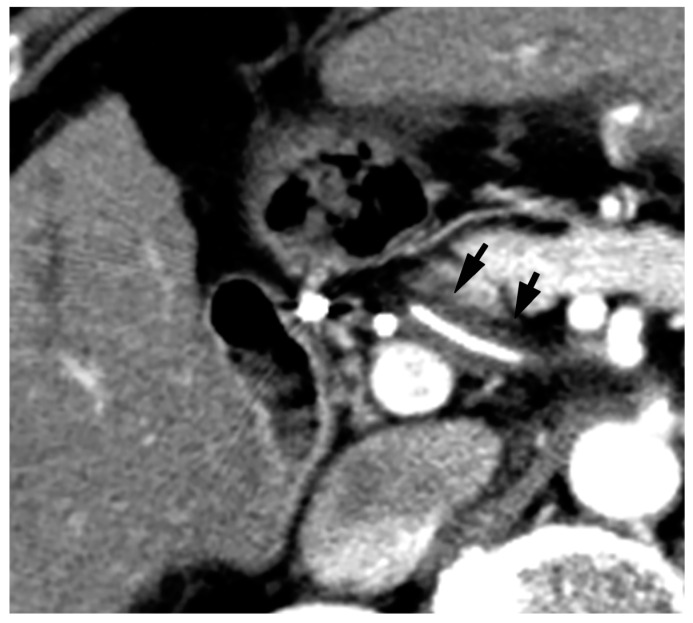
Arterial wall thickening (arrows) observed around the catheter which is inserted into the common hepatic artery for hepatic arterial infusion chemotherapy for the treatment of metastatic liver cancer.

Since BDN represents a precursor lesion to biloma, intrahepatic biloma formation sometimes coexists with BDN. In such cases, the abovementioned linear hypodense structure, along with the portal tract, connects with the biloma. Advanced BDN can lead to portal vein thrombosis (Figure 10), and may cause segmental staining associated with portal vein perfusion disorders on the arterial phase of contrast-enhanced CT images. After repeated HAIC, BDN cases may show a thickening of the arterial wall and elevated fat density around the artery with catheter insertion, such as the common hepatic artery or proper hepatic artery, which is a finding of hepatic arterial damage caused by HAIC (Figure 11) [29].

• Ultrasound (US) findings of bile duct necrosis

BDN shows similar findings to intrahepatic bile duct dilatation on both pre-contrast CT and CE-CT, but this structure is “peribiliary necrotic tissue” rather than a “fluid-filled dilated duct” and it is not depicted on US as an echo-free duct-like structure.

**Figure 12:**
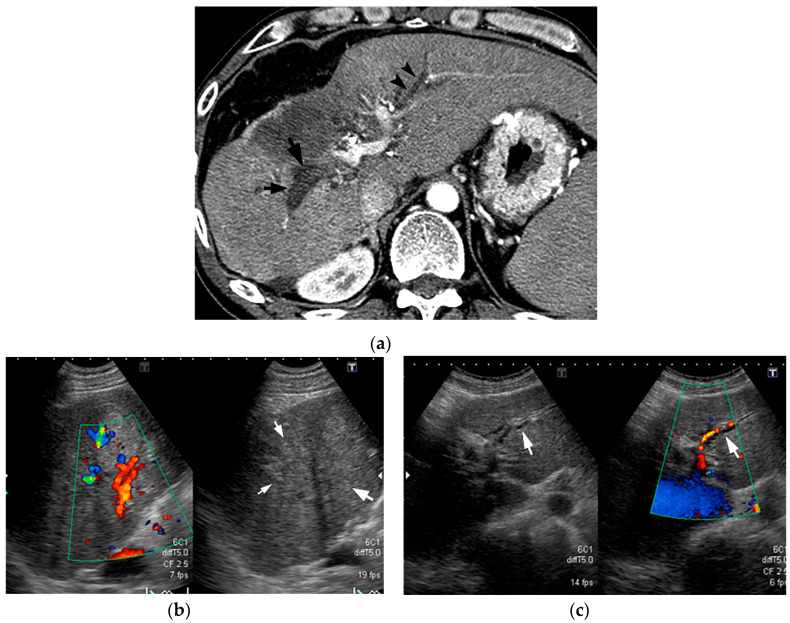
Comparison between CT and ultrasound (US) findings of BDN. (**a**) On contrast-enhanced CT, a hypodense thick tubular structure (arrow) is observed in the hepatic hilum of the right hepatic lobe, and in the left lateral segment, a similar hypodense thin tubular structure (arrowheads) is observed. Although these tubular hypodense structures look like dilated intrahepatic bile ducts, the right lobe lesion is BDN and the left lateral lobe lesion is bile duct dilatation. (**b**) On US images of the right hepatic lobe, no dilated bile ducts are observed in the portal tract. The portal tract shows iso- to slightly hyper-echo compared to the surrounding liver parenchyma (arrows). This US finding is inconsistent with the CT finding. (**c**) In the US image of left lateral lobe of the liver, a slightly dilated echo-free tubular structure (arrows) is observed along the arterial branch, which represents intrahepatic bile duct dilatation. As indicated on Figure 12b, BDN is iso- to slightly hyper-echo compared to the surrounding liver parenchyma and shows discrepancy with the CT finding.

In BDN, the damaged larger portal tracts are observed as slightly hyperechoic structures without a dilated intrahepatic bile duct (Figure 12) [9]. It is important to recognize the differences between CT and US findings for correct diagnosis of BDN.

• T2-weighted MR findings of bile duct necrosis

**Figure 13:**
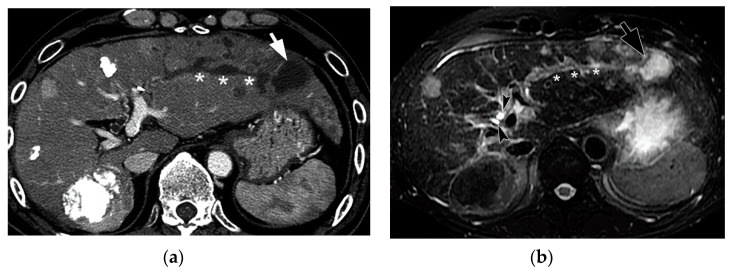
Comparison between CT and T2-weighted MRI findings of BDN. (**a**) On contrast-enhanced CT, a hypodense irregular tubular structure (*) is observed in the left lateral segment of the liver. Although this tube-like structure looks like irregularly dilated intrahepatic bile ducts, it is BDN. Biloma formation (arrow) is observed at the peripheral portion of the lateral segment which is connected to BDN. On the hepatic hilum, slightly dilated right and left hepatic ducts are observed (arrowheads). (**b**) On T2-weighted MRI, BDN (*) and biloma (arrow) on the left lateral lobe of the liver do not show marked hyperintensity like the hepatic ducts (arrowheads), but instead show slight hyperintensity. This finding suggests that BDN and biloma are not pure fluid-filled spaces, but “necrotic tissue”. (Figure 13a is same figure as Figure 9. It is presented for comparison with Figure 13b).

**Figure 14:**
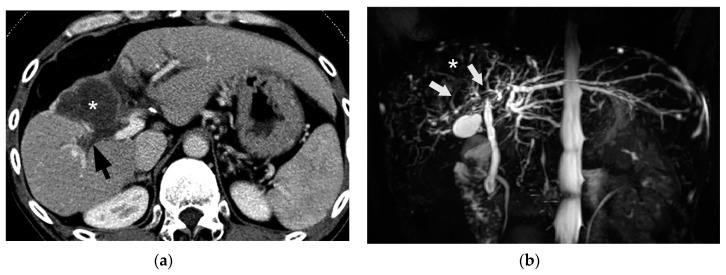
Comparison between CT and MR cholangiopancreatography (MRCP) findings of BDN. (**a**) On contrast-enhanced CT, a hypodense tubular structure is observed in the hilar area of the right liver lobe (arrow). Adjacent to this tubular structure, a large hypodense mass is observed in the medial segment of the liver (*). These structures are BDN (arrow) and a biloma (*). A dilated intrahepatic bile duct is also observed in the lateral segment of the liver (arrowheads). (**b**) On MRCP, although dilated intrahepatic bile ducts in the left lateral lobe of the liver are clearly observed, bile ducts in the hilar region and right hepatic lobe are unclear (arrow). Furthermore, the biloma in the medial segment of the liver is not clearly depicted on MRCP (*).

On T2-weighted MR images, BDN does not show marked high intensity, but slightly high intensity, which represents portal tract damage caused by imbibed bile salt, rather than a fluid-filled dilated bile duct (Figure 13). On MR cholangiopancreatography (MRCP), a necrotic portion of BDN shows variable signal intensities depending on the ratio of water to necrotic tissue (with/without hemorrhage) contents on heavy-T2 weighted images, which reflects the absence of intrahepatic bile duct dilation at the BDN site (Figure 14). Recognizing the difference between the T2-weighted MR images and CT findings provides confidence in making a diagnosis of BDN and not intrahepatic bile duct dilatation.

• Hepatocyte-specific MR contrast agent and bile duct necrosis

**Figure 15:**
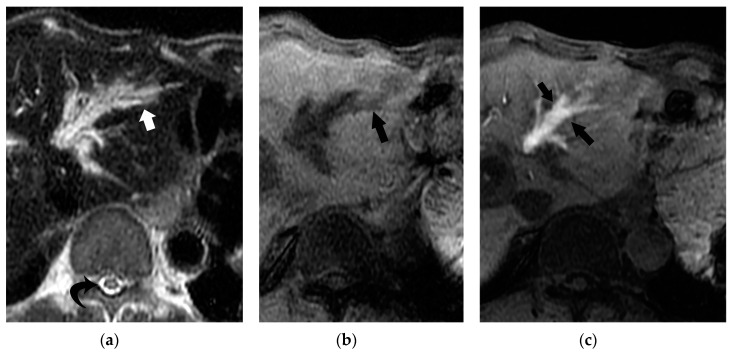
MR findings of BDN. (**a**) On T2-weighted MRI, BDN (arrow) on the left lateral lobe of the liver shows thick tubular hyperintensity, and the degree of hyperintensity is not so high compared to the cerebrospinal fluid observed in the spinal canal (curved arrow). (**b**) In the hepatobiliary phase of Gd-EOB-DTPA (EOB), which is 20 min after intravenous (iv) administration, the BDN site shows hypointensity compared to the background liver (arrows). Usually, in this phase, the intrahepatic bile duct shows hyperintensity because of secretion of EOB to the bile. (**c**) About 70 min after intravenous administration of EOB, the BDN area shows marked hyperintensity (arrow), which represents EOB exuding into the BDN region and being retained at the necrotic area.

Gd-EOB-DTPA (EOB) is a hepatobiliary MR contrast agent for T1-weighted imaging that is taken up by the organic anion transporters OATP1B1/B3 into hepatocytes and excreted through the biliary pathway according to the efficiency of each hepatocyte [38,39]. About 20 min after intravenous administration, EOB is taken up by hepatocytes, and the hepatic parenchyma shows hyperintensity and a liver lesion which consists of cells/tissue without hepatocellular origin which shows hypointensity because of no uptake of EOB in this area. Interestingly, in BDN, EOB gradually oozes out through the necrotic bile duct towards the surrounding periductal necrotic tissue, and the necrotic area shows marked hyperintensity on the super-delayed phase (70 min after intravenous injection) (Figure 15). The super-delayed phase image of Gd-EOB-DTPA on enhanced MRI might have potential to clearly depict the bile duct necrosis area by the exudation of EOB toward the damaged liver tissue [40].

**Figure 16:**
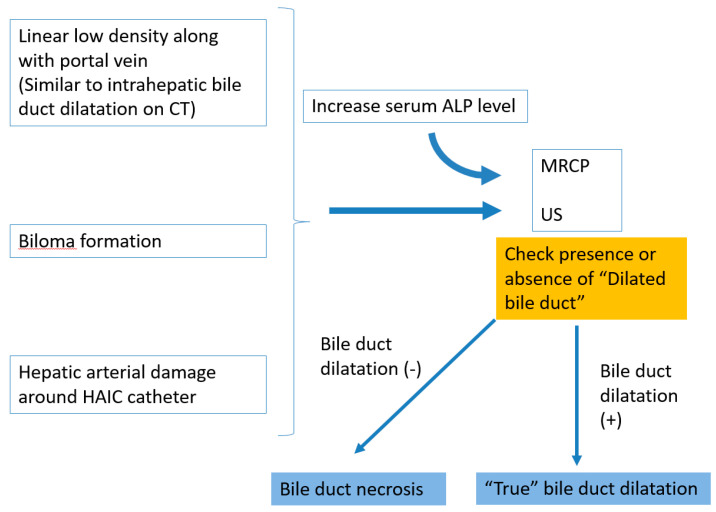
Diagnostic algorithm of BDN during the course of repeated transcatheter chemoembolization therapy (TACE) or hepatic arterial infusion chemotherapy (HAIC) for treatment of a liver tumor.

The diagnostic algorithm of BDN during the course of repeated TACE or HAIC for treatment of a liver tumor is shown on Figure 16.

## 6. Progression of Bile Duct Necrosis on Sequential CT Images

Because of the chemical toxicity of the imbibed BAs, BDN, including the surrounding liver damage, progresses even after the discontinuance of the TACE/HAIC. For example, periductal tissue damage progresses persistently, and about 90% of the cases show portal vein obstruction adjacent to BDN [36]. Disruption of portal vein blood flow leads to hepatic atrophy in the portal perfusion area.

## 7. Mimickers of “Bile Duct Necrosis” on Imaging

In this section, we review the disease and pathological conditions that may show similar imaging findings to that of BDN.

• Portal vein thrombosis

**Figure 17:**
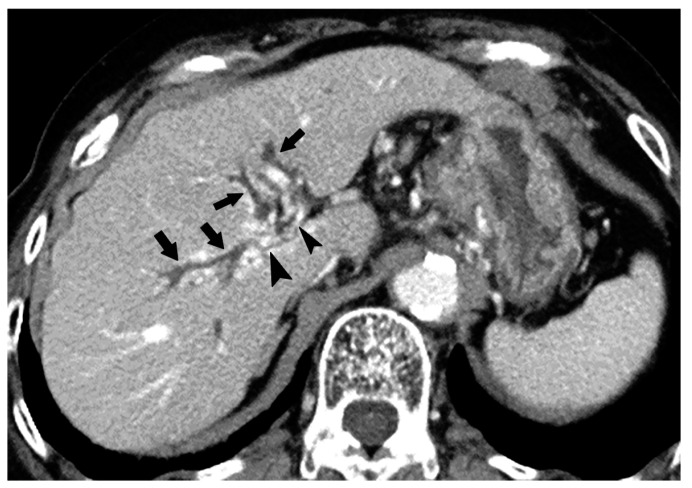
Intrahepatic portal vein thrombosis in an 84-year-old woman. In the portal venous phase of contrast-enhanced CT, the portal vein in the hepatic hilum shows linear hypodensity (arrows) and looks like bile duct dilatation. However, there are markedly enhanced multiple vessels (arrowheads) around the hypodense linear structure, which is called cavernous transformation of the portal vein. It is a typical finding of portal vein thrombosis in the hepatic hilum area.

In adult patients, cirrhosis, hepatic tumor, hypercoagulable state, pancreatitis, inflammatory bowel disease, myelo-proliferating disease, and the post-abdominal surgery state are the main causes of portal vein thrombosis [41,42]. The thrombotic portal venous branch within a large portal tracts show tubular hypodense structures on CE-CT and might show findings that mimic BDN (Figure 17). In portal vein thrombosis, cavernous transformation of the portal vein is sometimes observed in portal vein thrombosis of the hepatic hilum area, especially in the chronic phase of portal vein thrombosis [43]. On arterial phase images on CE-CT, hepatic parenchyma, which is nourished by the thrombosed portal venous branch, shows segmental staining in portal vein thrombosis. Such segmental staining on the arterial phase image of CE-CT is generally not observed in BDN. However, in advanced BDN, secondary portal vein thrombosis might occur by toxic detergent reaction of BAs which infiltrate into the portal venous wall. In such cases, BDN also shows segmental staining on the arterial phase of CE-CT.

• Obstructive jaundice (bile duct dilatation)

**Figure 18:**
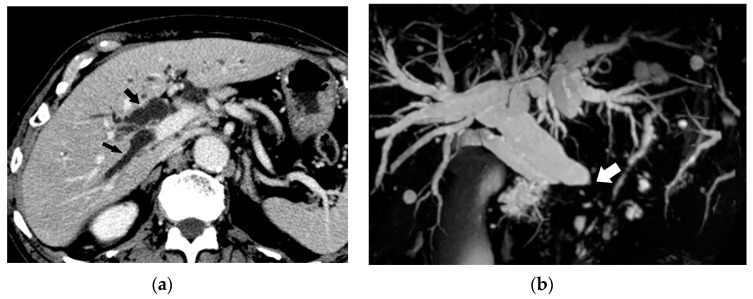
Obstructive jaundice (intra- and extra-hepatic bile duct dilatation) observed in a 77-year-old man. (**a**) In the portal venous phase on contrast-enhanced CT, a dilated linear hypodense structure (arrows) is observed along the portal vein in the hepatic hilar area. (**b**) On MRCP, common bile duct obstruction caused by common bile duct carcinoma is observed (arrow). The upstream dilated bile ducts gradually taper as they move toward the peripheral part of the liver.

Biliary obstruction caused by a tumor/stones in the lower biliary system blocks bile flow and shows bile duct dilatation in the upper part of the bile duct [44]. On pre-contrast CT and CE-CT, such dilated bile ducts observed in obstructive jaundice cases show hypointense tubular structures similar to BDN (Figure 18). However, usually in obstructive jaundice cases, it is easy to identify the cause of biliary obstruction, such as a common bile duct stone or a common bile duct carcinoma, on imaging in the downstream of the dilated bile duct with CT, MRI, and MRCP. In obstructive jaundice cases, the dilated bile duct is easily recognized on US and MRCP. In contrast, in BDN cases, the hypodense tubular structure observed on CT generally does not show typical findings of bile duct dilatation on US and MRCP. Another important finding which can differentiate BDN and bile duct dilatation might be the extent of the hypodense tubular structure on CT. In BDN, generally, the hypodense tubular structure on CT is restricted to the necrotic area of the biliary tract. In contrast, in obstructive jaundice cases, hypodense tubular structures on CT are observed throughout the bile duct upstream of the biliary obstruction site and gradually taper off as they move toward the peripheral part of the liver.

• Peribiliary cyst

**Figure 19:**
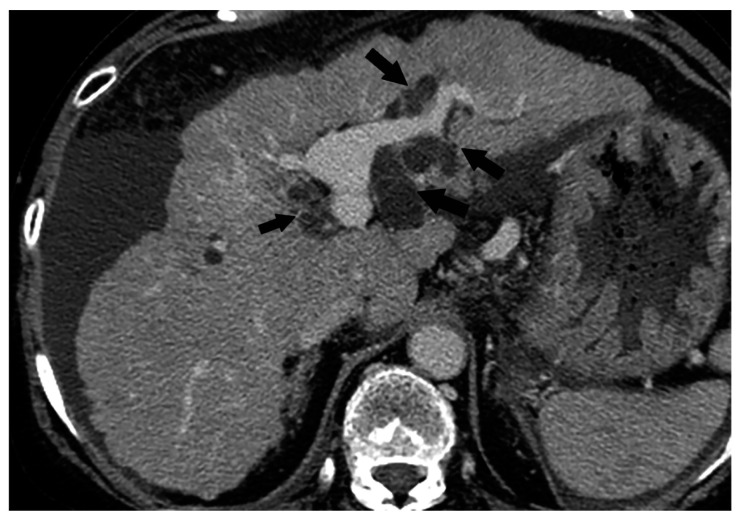
Peribiliary cyst observed in a 56-year-old man with cirrhosis. In the portal venous phase on contrast-enhanced CT, dilated multiple hypodense cystic structures (arrows) are observed along the left main portal vein and its branch in the hepatic hilar area. These structures are peribiliary cysts, showing cystic dilatation of the peribiliary glands around the large bile duct. Although this hypodense structure looks like intrahepatic bile duct dilatation, it is located bilaterally along the portal vein, unlike dilated bile duct (arrows).

Peribiliary cyst (PC) is a cystic dilatation of the peribiliary glands outside the intrahepatic large bile duct wall. PC is associated with polycystic liver and cirrhosis at a high rate [45]. It is also present in other chronic liver diseases, portal hypertension, and ductal plate malformation. PC observed in cirrhotic cases generally increases in number and size as the cirrhosis progresses. PC shows hypodensity, and a well-defined intrahepatic structure around the liver hilum on pre-contrast CT and CE-CT (Figure 19). PC shows marked hyperintensity on T2-WI MRI given its retention of cysts of the peribiliary glands. Although intrahepatic bile duct dilation is observed only on one side along the portal vein, PCs are located bilaterally along the portal vein (Figure 19).

• Caroli’s disease

**Figure 20:**
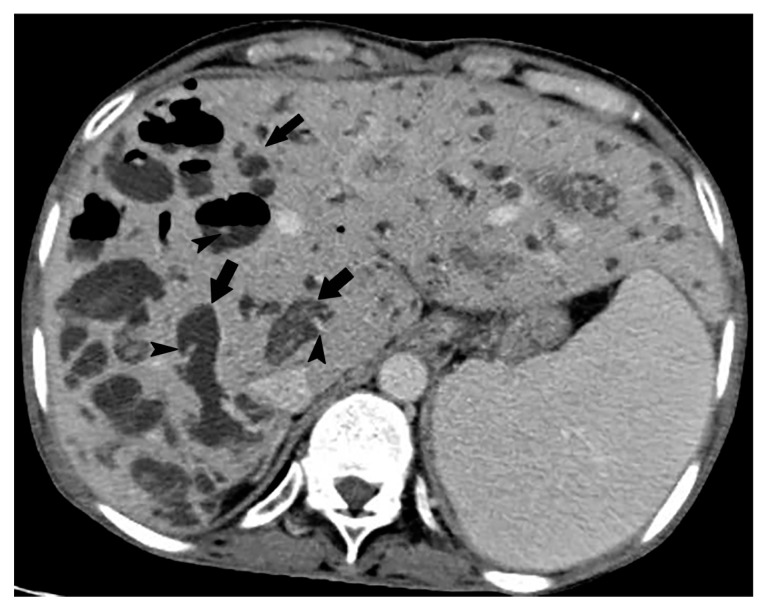
Caroli’s disease in a 58-year-old man. In the equilibrium phase on contrast-enhanced CT, multiple hypodense cystic or tube-like structures (arrows) are observed throughout the liver. Contrast-enhanced portal venous branches (arrowheads) are observed in the center of the hypodense structure, which is called the “central dot sign”, a characteristic finding of Caroli’s disease.

Caroli’s disease (CD) is a rare congenital disorder of bile duct formation that develops as non-obstructive cystic dilation of the intrahepatic bile duct system [46]. CD shows autosomal recessive inheritance. Abnormal cystic dilatation of bile ducts in CD may distribute in a regional or diffuse manner. On CT, the lesion shows multiple hypodense rounded areas which are inseparable from the dilated intrahepatic bile ducts (Figure 20). The contrast-enhanced portal venous branch within the dilated intrahepatic duct is referred to as the “central dot sign” [47].

**Figure 21:**
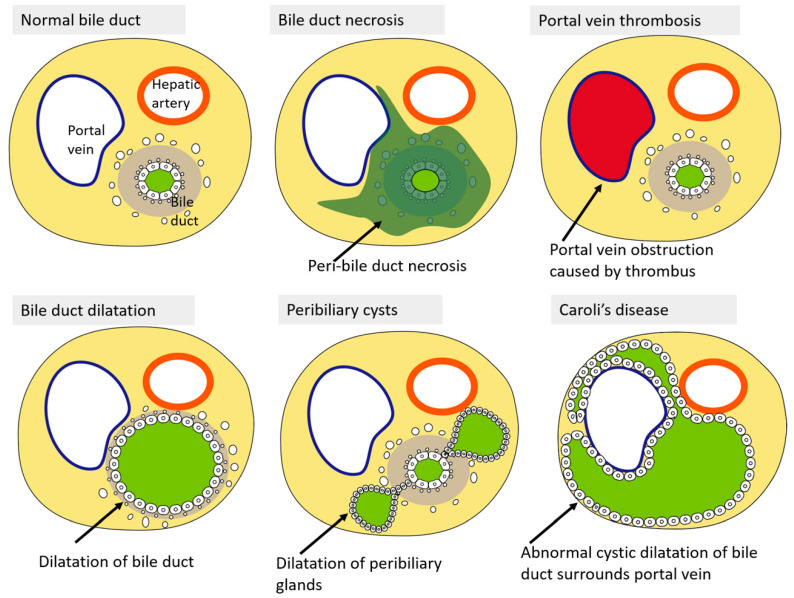
Schematic diagram of the pathological characteristics which show a hypodense tube-like structure on pre-contrast-enhanced CT and contrast-enhanced CT in BDN and its mimickers.

Schematic diagram of the pathological characteristics of BDN and its mimickers are shown on Figure 21.

## 8. How to Treat Bile Duct Necrosis

BDN is a preceding stage of overt biloma, also known as a branched-type biloma [8,12]. If BDN is detected on imaging during the course of repeated TACE or HAIC for treatment of a liver tumor, we should consider such cases as at high risk of overt biloma formation. In such cases, no additional TACE or HAIC should be performed [10]. When biloma occurs, conservative treatment is appropriate unless there are symptoms or interval increase in the biloma [10]. However, in 20–65% of biloma cases, symptoms such as mild to severe signs of infection or interval increase in the biloma are seen [10,11,12].

**Figure 22:**
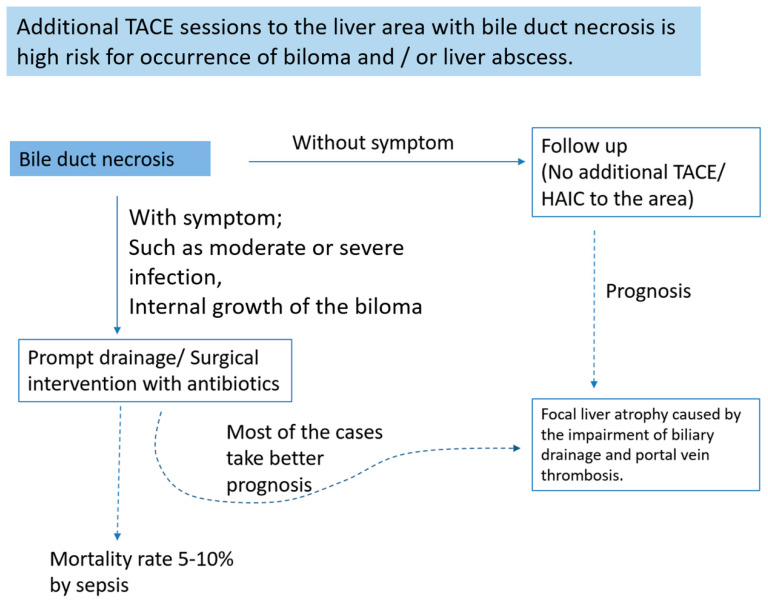
Schematic diagram of the treatment strategy for BDN.

Even in symptomatic cases, the outcome of most intrahepatic biloma is not poor if prompt percutaneous drainage or surgical treatment is performed in addition to the use of antibiotics. The mortality rate of patients with biloma formation after TACE is about 5–10% [11,12]. Therefore, early detection of BDN and biloma on imaging and careful treatment might be mandatory to improve patient prognosis with repeated TACE or HAIC for liver tumors (Figure 22). Once infection of the biloma is suspected, prompt appropriate treatment should be considered to avoid treatment-related death [10].

## 9. Conclusions

BDN and biloma formation is one of the serious complications of transcatheter therapy for hepatic malignancy. Ischemic injury of the PBP, the supportive vessel of the bile duct epithelium, is intimately associated with the formation of BDN and subsequent development of biloma. Once BDN occurs, persistent tissue damage to the surrounding bile duct is induced by imbibed bile juice, and portal vein thrombosis may also be observed. Differentiation of the imaging findings of CT and US, MRI, and MRCP is important to distinguish “bile duct necrosis” from “bile duct dilatation”. Although most cases of BDN and biloma can be treated conservatively, if there are some symptoms such as moderate or severe infection or interval growth of the biloma, prompt treatment is essential to avoid lethal abscess formation and sepsis.

## Figures and Tables

**Table 1:** Bile duct epithelium protection mechanisms from the aggressive bile acids (BAs).

Mechanism	Notes
Cholehepatic shunt pathway	Bile duct epithelium absorbs BAs through the BA transporter and excretes them into the peribiliary vascular plexus. They are then transported to hepatocytes
Mucin secretion from peribiliary glands	Limited to large bile ducts which have peribiliary glands around the duct wall
Bicarbonate secretion by bile duct epithelium (HCO_3_^−^ umbrella)	Limited to small bile ducts
Phospholipid secretion from hepatocytes	May depend on hepatocyte functional state
